# A case report of high-grade intraepithelial neoplasia of the bronchial mucosa

**DOI:** 10.1186/s12890-024-03220-5

**Published:** 2024-08-29

**Authors:** Xuefeng Li, AiMin Sun, Xuefei Bai, Zongtao Hu, Yin He

**Affiliations:** 1 Department of Thoracic Oncology, Hefei Cancer Hospital, Chinese Academy of Sciences, No. 68 Yangqiao Road, Hefei, 230000 China; 2 Department of Respiratory, Fuyang People’s Hospital, No.501 Sanqing Road, Fuyang, 236000 China; 3 Department of Pathology, Hefei Cancer Hospital, Chinese Academy of Sciences, No. 68 Yangqiao Road, Hefei, 230000 China

**Keywords:** Interventional bronchoscopy, Squamous epithelium, High-grad intraepithelial neoplasia, Case report

## Abstract

**Background:**

Lung cancer is the most common cause of cancer death worldwide and poses an immediate health threat. Despite decades of basic and clinical research, the 5-year survival rate for lung cancer patients is less than 10%.The most important drawbacks in efficient treatment of lung cancer are delayed diagnosis and absence of effective screening. Detection and study of precancerous lesions of the bronchial mucosa might be one of the turning points in understanding of neoplastic transformation. Therefore, it would be the most effective prevention and early treatment modality. We report a case of high-grade intraepithelial neoplasia of the bronchial mucosa in which a neoplastic growth in the lumen of intrinsic segment in the upper lobe of the left lung was detected on electronic bronchoscopy, and biopsy confirmed squamous papillary hyperplasia with high-grade intraepithelial neoplasia.

**Case presentation:**

A 74-year-old male was admitted to the hospital due to a mass lesion in his left lung. After admission, computed tomography scan of the chest showed an intraluminal mass in the intrinsic segment of the upper lobe of the left lung and an enlarged left hilum.

**Conclusions:**

High-grade intraepithelial neoplasia of the bronchial mucosa is rare in the respiratory system. We report a case that can provide useful information for early diagnosis and treatment of the disease.

## Background

High-grade intraepithelial neoplasia of bronchial squamous epithelium(high-grad intraepithelial neoplasia, HGIN)was described in the 90s of the 20th century. The World Health Organization (WHO) classifies pre-invasive squamous lesions into nine categories, ranging from normal (A) to invasive cancer (I), as shown in Table 1 [1]. The risk and timeline for progression of bronchial intraepithelial lesions to carcinoma in situ (CIS) or invasive carcinoma are quite clear [2]. The latest edition of the WHO classification of lung neoplasms classifies squamous epithelial atypical hyperplasia and carcinoma in situ as a precursor lesion to squamous cell carcinoma, whereas in the 2015 edition of the WHO classification it is referred to as a preinvasive lesion [3]. Squamous cell precursor lesion is precancerous lesions of squamous lung cancer and is a transitional stage in the progression of the lesion to squamous lung cancer.The causes of lung precancerous lesions are multifactorial, and clinically patients usually show no symptoms, while some patients with lung precancerous lesions show symptoms of dyspnea, hemoptysis, cough, sputum, runny nose and fever [4–6]. Autofluorescence bronchoscopy (AFB) and narrow band imaging (NBI) are more sensitive than white light bronchoscopy (WLB) in detecting and evaluating precancerous lung lesions [7]. Surgery is the gold standard for treating precancerous lesions [4]. Intraluminal bronchoscopic treatment such as electrocautery, argon plasma coagulation, Nd-YAG laser, photodynamic therapy, and cryotherapy are an option for patients who are not candidates for surgical treatment [2]. Here we report a case of high-grade intraepithelial neoplasia of the bronchial mucosa diagnosed by transbronchoscopic biopsy, which had not been previously reported.

**Table 1:** The World Health Organization (WHO) classifies pre-invasive squamous lesions into nine categories

	Histologic grade
A	Normal
B	Inflammation / bronchitis
C	Hyperplasia
D	Squamous metaplasia
E	Low grade dysplasia
F	Moderate dysplasia
G	High grade dysplasia
H	Carcinoma in situ
I	Invasive carcinoma

## Case presentation

### Chief complaints

A 74-year-old man was admitted to the clinic with complaints of cough after symptoms of nausea and vomiting.

### History of present illness

Left lung mass lesions were not absorbed after anti-inflammatory treatment at a local hospital.

### History of illness

The patient had a herniated lumbar disc for 5 years. The patient had multiple cerebral infarctions for 5 years.

### Personal and family history

The patient denied any occupational history of exposure to organic or inorganic dust. The patient has a long history of smoking for 40 years (one packet of cigarettes per day). The patient denied any family history of related conditions.

### Physical examination

Physical examination revealed left breath sounds were reduced, right lung breath sounds were clear, and dry and wet rales were not heard.

### Laboratory examinations

Serum carcinoembryonic antigen (CEA) was 1.06 ng/mL, squamous cell carcinoma antigen(SCCA) was 0.68 ng/mL, neuron specific enolase (NSE) was 5.31ng/mL, cytokeratin fragment antigen21 -1(Cyfra21-1) was 2.43ng/mL, and serum pro-gastrinreleasing peptide (Pro-GRP) was 51pg/mL.

### Imaging examinations

**Fig. 1:**
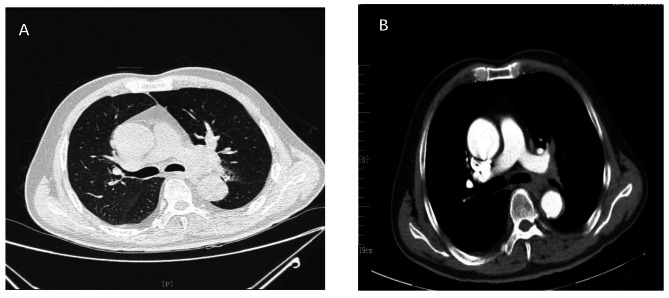
(**A**-**B**) Chest computed tomography scan on 2023.09.13. **A**: Lung window; **B**: Mediastinum windows

Computed tomography (CT) scan of the chest showed the intrinsic segment of the upper lobe of the left lung has an intraluminal mass and the left hilum was enlarged (Fig. 1).

### Futher diagnostic workup

**Fig. 2:**
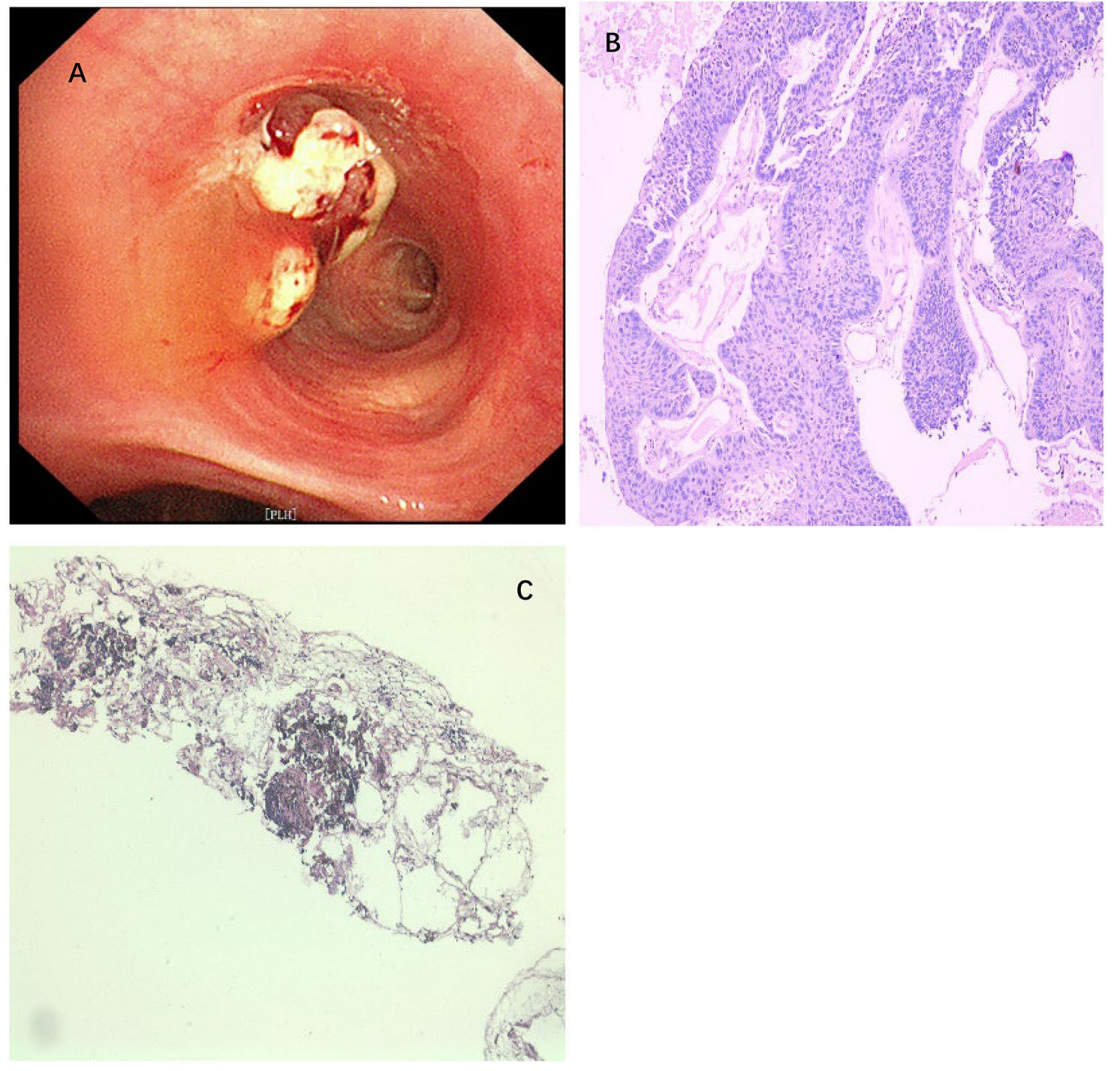
**A**: New organisms under the first tracheoscopy; **B**: Pathology of new biological biopsy in the lumen of the bronchi (Hematoxylin-eosin staining; Magnification: × 10); **C**: Pathological biopsy of 11, left interlobar (Hematoxylin-eosin staining ; Magnification:× 10)

After white light bronchoscopy (WBL) detection, an endobronchial cryobiopsy of intrinsic segment in the upper lobe of the left lung was performed. Endoscopic examination showed new biological obstruction of intrinsic segment of the upper lobe of the left lung. Lymph node metastasis had to be excluded (Figure 2).

The biopsy results. A: Multiple papillary space-occupying masses visible during tracheoscopy; B: Squamous papillary hyperplasia with atypic cells involving the full thickness of the epithelium; C: Pathological biopsy of 11, left interlobar revealed extrusion of deformed lymphocytes (Fig. 2).

### Final diagnosis

Based on the results of the biopsy and pathological examination, the final diagnosis was high-grade intraepithelial neoplasia of bronchial squamous epithelium.

### Treatment

**Fig. 3:**
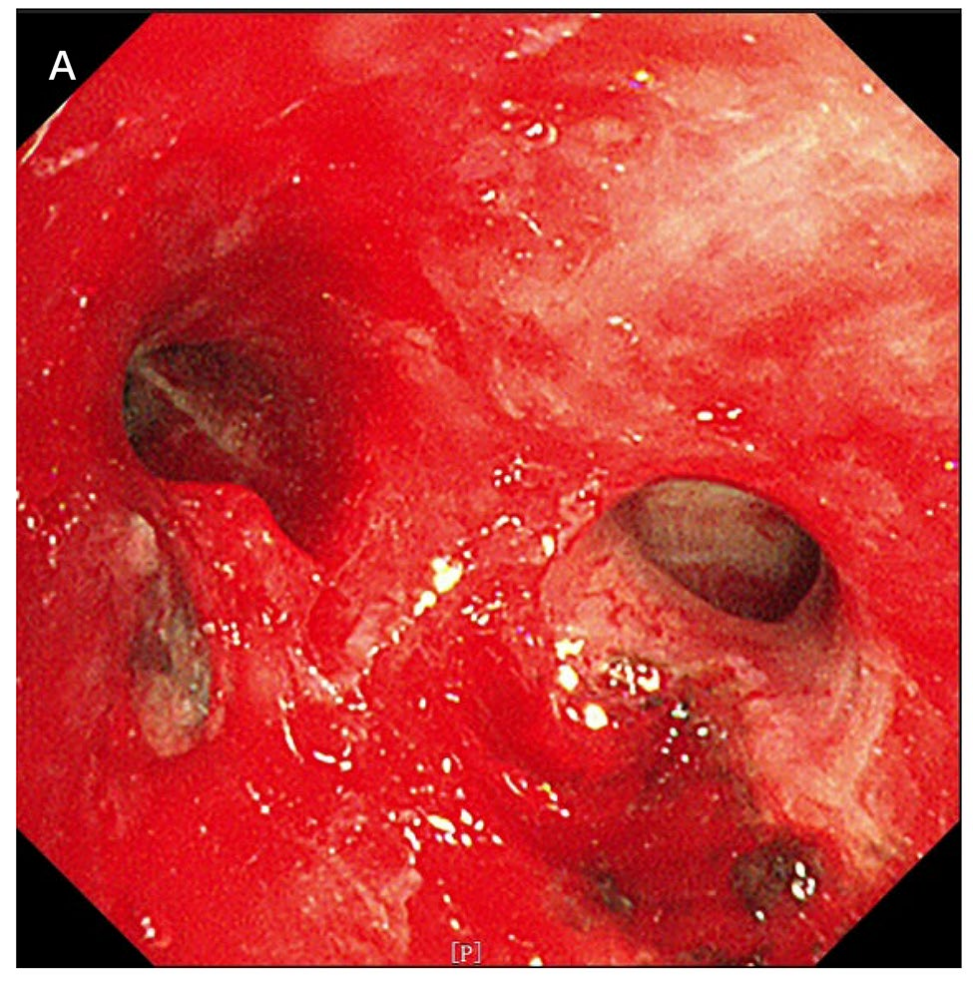
**A**: After tracheoscopic tumor elimination, the intrinsic segment lumen of the upper lobe of the left lung was unobstructed

Dure to lumen obstruction of intrinsic segment of the upper lobe of the left lung, endoscopic resection of bronchial lesions was performed to ensure the basic structure and patency of the airway.In brief, under tracheoscopy we performed multiple new biological CO2 freezing, electrocautery snare and high-frequency electrocautery to stop bleeding (Fig. 3).

### Outcome and follow-up

**Fig. 4:**
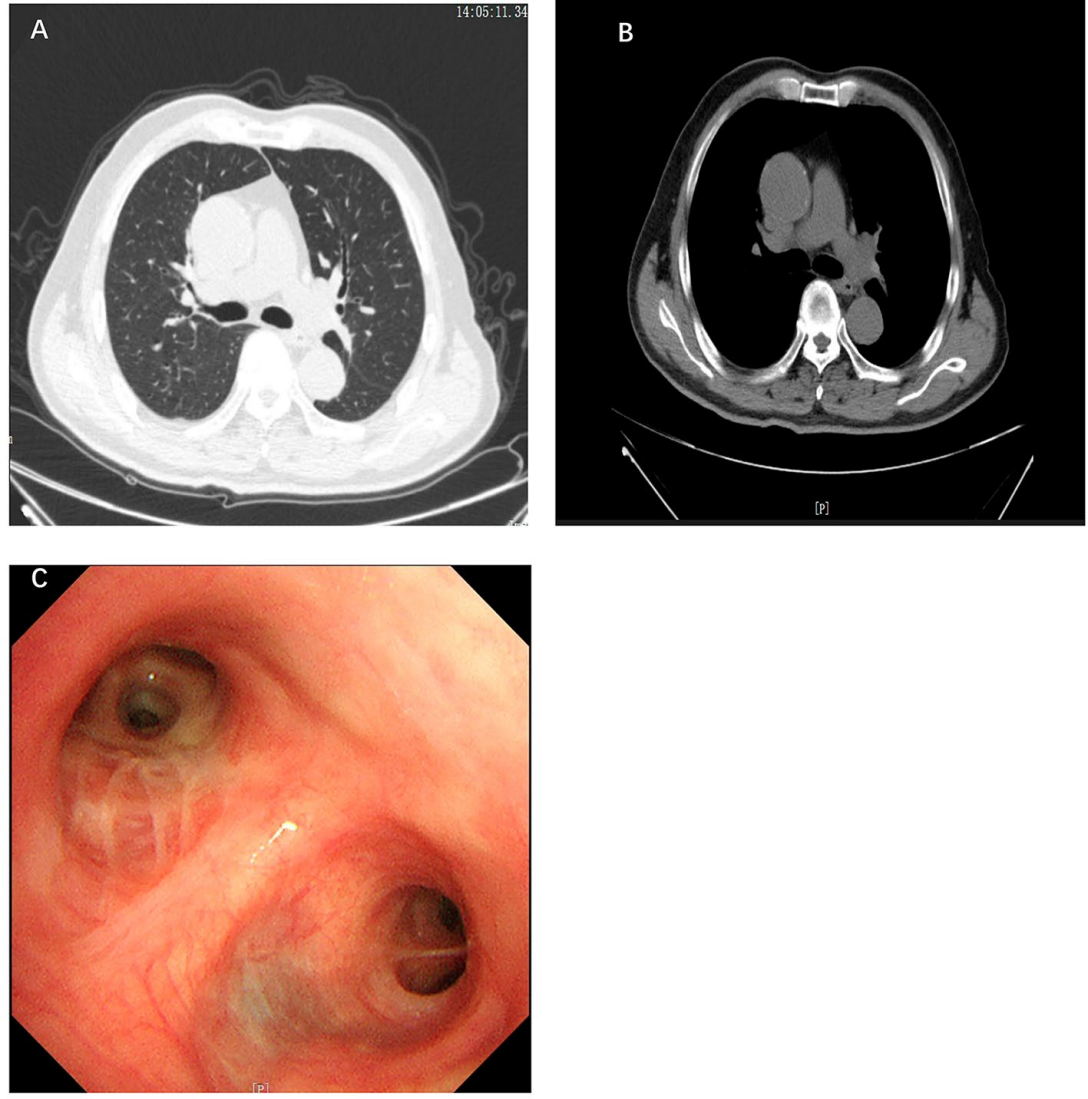
(**A**-**B**) Repeat chest computed tomography scan on 16 October 2023. **A**: Lung window; **B**: Mediastinum windows; **C**: Tracheoscopy was reviewed on 11 October 2023

**Fig. 5:**
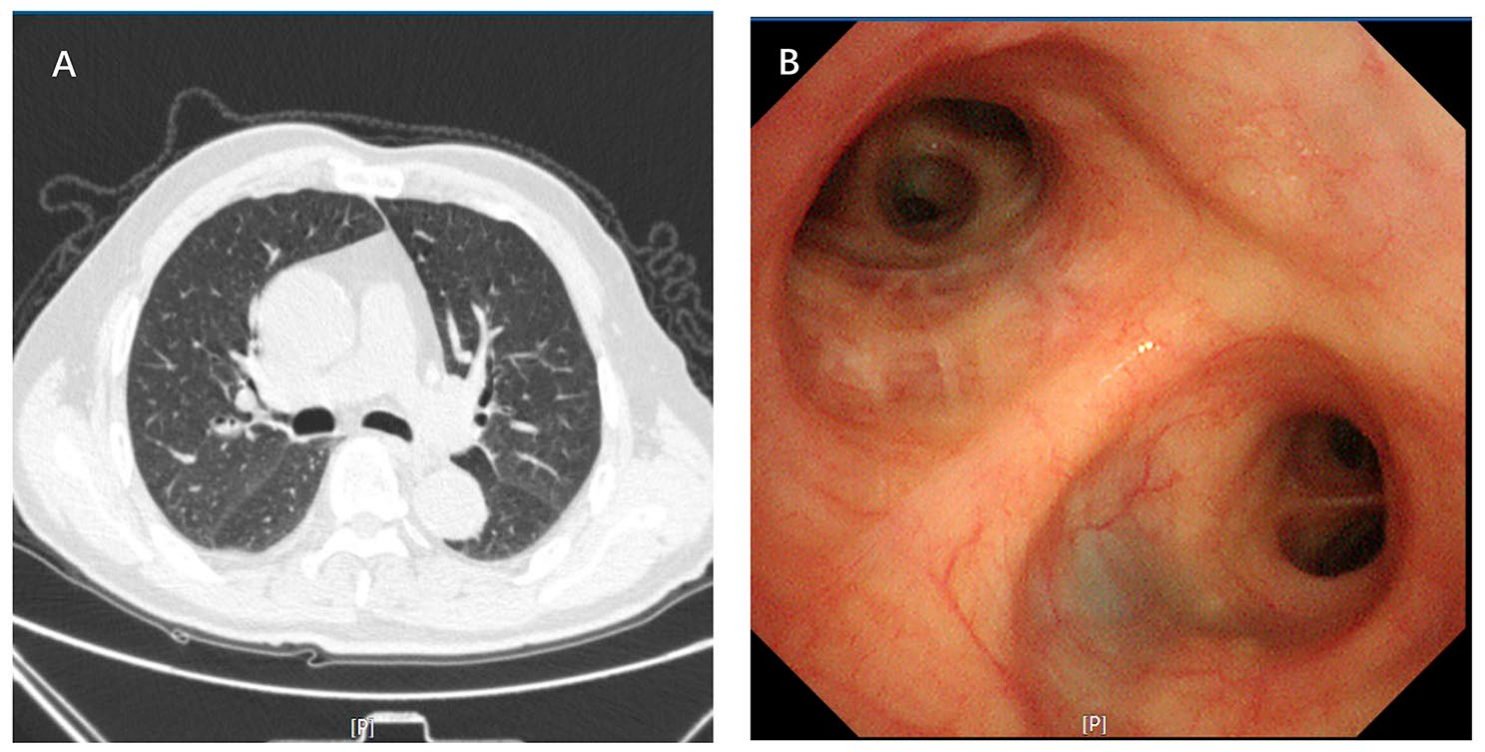
**A**: Repeat chest computed tomography scan on 20 February 2024; **B**: Tracheoscopy was reviewed on 21 February 2024

The patient recovered well without any discomfort. At one-month follow-up, the patient’s repeat tracheoscopy and chest computer tomography (CT) showed that the intrinsic segment lumen of the upper lobe of the left lung was patency and the mucosa was smooth (Fig. 4). At three-month follow-up, the patient’s left upper lobe lesion was stable by repeat bronchoscopy and chest computed tomography (CT) scans (Fig. 5).

## Discussion

In 1960 Richard first proposed the concept of intraepithelial neoplasia, a precancerous change in the squamous epithelium of the cervical mucosa [8]. Richard suggested the lesions that had the features of cancer precursors were regarded as high-grade cervical intraepithelial neoplasia [9]. The WHO’s classification of lung precancerous tumors describes changes in the grading of bronchial lining cells, from normal epithelium to increased grade dysplasia to carcinoma in situ (CIS) and invasive carcinoma [10]. CIS may occur alone and independently, or it may be an orthotopic spread of adjacent invasive lung cancer [11]. The sequence of precursor lesions for squamous cell carcinoma may be hyperplasia-metaplasia-dysplasia-CIS.Not all squamous cell carcinoma in situ (SCIS) progresses to invasive disease and almost one third to half may either regress or remain stable [12]. This progression of disease has long been recognized.

It is well recognized that the use of white light bronchoscopy in detection of precancerous lesions yields has low sensitivity and specificity. The introduction of narrow-band imaging (NBI) and autofluorescence imaging (AFI) bronchoscopy into the diagnostic evaluation of lung cancer has significantly increased the sensitivity of detection of precancerous lesions.At the same time, clinical studies had found that the use of AFI(autofluorescence fluorescence imaging)and NBI (narrow-band imaging) had good potential for detection of precancerous bronchial lesions [13]. The results of one study suggested that combining strategy of AFB and WLB would be more effective than AFB and WLB alone in diagnosing high-grade lesions when evaluating premalignant airway lesions [14]. NBI has high sensitivity and specificity in the diagnosis of precancerous airway lesions and is superior to AFI. However, combining AFI and NBI does not significantly improve the diagnostic efficiency of airway neoplasia at a preinvasive stage [15]. Preinvasive lesions, such as CIS, are usually small, limited to the bronchial wall, and difficult to visualize using conventional white light bronchoscopy [16]. In our case, Mass lesions of the intrinsic segment of the upper lobe of the left lung were seen by white light bronchoscopy, and according to previous reports, most patients were rarely diagnosed with preneoplastic lesions.

High-grad intraepithelial neoplasia is the pre-invasive stage of squamous cell carcinoma. About the treatment of the disease, although respiratory interventional techniques like photodynamic therapy (PDT), cryotherapy, mechanical debulking with biopsy forceps, electrocautery and argon plasma coagulation (APC) are becoming more sophisticated, surgery is still the gold standard for the treatment of CIS. In our case, because the patient’s lung lesion was close to the left hilum, the left lung would be resected completely. The patient refused surgery and we opted for respiratory endoscopic intervention like bronchoscopic cryotherapy, electrocautery, and mechanical debulking with CO2 cryotherapy. At the same time, bronchoscopy may replace surgery as first line recommended therapy for central SCIS and can be used as a reference for postoperative follow up [16].

Fiberoptic bronchoscopy is an important tool in the diagnosis of lung cancer [18]. Fiberoptic bronchial biopsy tissue is the gold standard for determining the nature of the lesion [7]. Bronchoscopic biopsies are diagnosed as squamous cell carcinoma when heterogeneous cells break through the basement membrane and infiltrate the entire layer under light microscopy, and conversely, they are classified as high-grade intraepithelial neoplasia of the squamous epithelium of the bronchial mucosa. In addition to this, we can use cytological methods to diagnose lung cancer, such as bronchial brush specimens and bronchoalveolar lavage (BAL) fluid specimens [19]. In our case, no cytological methods were added to help diagnose lung cancer. This is an under-reported aspect of our case.

## Conclusions

High-grade intraepithelial neoplasia of the bronchial mucosa is the pre-invasive stage of squamous cell carcinoma. We reported this case for the first time, providing some insight into its early detection and management. Our report does not support the general use of bronchoscopy as a screening tool for lung cancer, but suggest that it may be of use in certain groups.

## Data Availability

Data is provided within the manuscript or supplementary information files.
